# Characterization of the Biochemical Potential of Moroccan Onions (*Allium cepa* L.)

**DOI:** 10.1155/2022/2103151

**Published:** 2022-03-09

**Authors:** Amal Brahimi, Mohamed El Ouardi, Amal Kaouachi, Abdelhakim Boudboud, Lhoussain Hajji, Hassan Hajjaj, Hamid Mazouz

**Affiliations:** ^1^Biotechnology and Valorization of Biological Resources Laboratory, Faculty of Science of Meknes, University of Moulay Ismail, P.B. 11201 Zitoune, Meknes, Morocco; ^2^Cluster of Competency “Agri-Food, Safety and Security” IUC VLIR-UOS, Moulay Ismail University, Marjane 2, BP, 298 Meknes, Morocco; ^3^Values, Society and Development Laboratory, Faculty of Letters and Human Sciences Ibn Zohr University, Agadir, Morocco

## Abstract

*Allium cepa* L. remains the most cultivated *Allium* species in Morocco and around the world. With the purpose of making the first biochemical characterization of Moroccan onions, several biochemical components have been measured in eleven onion ecotypes. Onions were collected as seeds from different geographical origins and cultivated in the same environment, to eliminate the influence of the environment on biochemical expression. Moisture, total phenols, flavonoids, antioxidant activity, total and reducing sugars, and sulfur dioxide were the biochemical properties of interest. Except for moisture, the eleven onion ecotypes revealed a highly significant variation in terms of the studied biochemical characters. The total phenol and flavonoid content ranged from 5.94 to 11.22 mg equivalent gallic acid/g dry weight and 0.67 to 1.52 mg equivalent quercetin/g dry weight, respectively. The antioxidant activity of the studied onions showed a strong correlation with the polyphenols (*R*^2^ = 0.7189), especially with the flavonoids (*R*^2^ = 0.8063). The sulfur dioxide content parted from 85.60 to 30.43 ppm when measured using the Monier-Williams distillation method. The current results show that there is no correlation between total sugars and reducing sugars. In conclusion, these findings present a clear biochemical profile of Moroccan onion ecotypes, as well as confirm, for the first time, the presence of a clear variation between the biochemical profiles of Moroccan onion ecotypes, which could be useful for future valorization programs.

## 1. Introduction

Onion is one of the oldest and most valuable cultures practiced in the world. Despite the high-cost investment, fund, price fluctuation, and the absence of sustainable income, onion remains the most cultivated culture on the planet [1]. It has a total cultivated area of 5.15 million hectares and an annual production of 99.5 million tons [2]. In Morocco, onion culture is the second most important crop after potatoes. During the 2019–2020 season, 29177 hectares of onion bulbs were planted, yielding an average of 30.17 tons/ha [2]. For the same season, the economic income from onion culture was estimated to be 480.22 million USD [3].

The presence of alk(en)yl cysteine sulfoxide, polyphenols, particularly flavonoids, and a high antioxidant capacity in the onion plant enhance onion health-promoting properties such as anticancer, antiplatelet, antithrombotic, and antibiotic [4–6].

Moreover, onions contain a variety of secondary metabolites, the most important of which are polyphenols [7–9]. These phytochemical compounds are available in onion at a high level, with higher antioxidant activity functions [10]. The flavonoids are the main group of phenolic compounds present in onions [11]. It could be divided into subgroups, such as flavones, flavanones, flavonols, isoflavones, flavanonols, flavanols, chalcones, and anthocyanins [10, 12]. The predominant flavonol compound in onion is quercetin. It is a potent antioxidative, with a high radical scavenging activity [13]. It also has an effect on plant disease resistance and bulb skin coloration. In fact, the color of the onion plant's yellow flesh to the brown bulb is related to the concentration of quercetin and its derivatives [8, 14].

Morocco's climate is characterized by the Mediterranean and southern climatic zones, in the northern coastal and the interior regions. This climatic separation results from the Atlas Mountains' location in the center of Morocco. The rainy season in the northern regions lasts from November to March, with an average of 50 mm to 100 mm of rain per month. The temperature ranges between 22 and 25°C in the summer and 10 and 12°C in the winter. However, temperatures in the Atlas Mountains, particularly at higher altitudes, are lower in the wet season than in coastal regions, but in the southern and interior regions, temperatures range from 25 to 30°C in summer and drop to less than 15°C in winter [15].

Because of the necessity of adaptation to geographic conditions, each Moroccan region has a distinct onion ecotype, with a distinct color, bulb shape, flavor, shelf life, and genotype. In a previous study, we detected the presence of a clear genetic diversity among 16 onion ecotypes, coming from 14 different regions [16].

The present study is aimed at investigating the biochemical composition and diversity of eleven Moroccan onion ecotypes. The characters of interest are polyphenols, flavonoids, total and reducing sugars, and sulfur dioxide content. In addition, moisture content and antioxidant properties were also measured. Thus, due to the scarcity of research on the biochemical profile of Moroccan onions, the current study could serve as a reference for the biochemical properties of dry onion bulbs in Morocco.

## 2. Material and Methods

### 2.1. Plant Material Collection

As part of the establishment of the first Moroccan onion ecotype collection, 11 onion ecotypes were collected as seeds in collaboration with the National Office of Agricultural Advices (ONCA) and the Agricultural Development Regional Office (ORMVA). It is important to state that the seeds were only collected from farmers who are seed producers. Following bulb harvest, eleven ecotypes were subjected to the present biochemical characterization. In the cultivated onions, five ecotypes have red bulb skin (P3, P4, P10, P16, and P17), one ecotype has white color (P2), and five have yellow skin color (P6, P7, P8, P9, and P18). The geographic origin of onion ecotypes varied between 61 m and 1442 m above sea level. During the 2018–2019 planting season, the average rainfall and temperature in onion origin areas ranged from 20.7 to 58.8 mm and 7 to 35°C, respectively (Table 1).

### 2.2. Field Experiment

The field experiment was conducted during the season 2018–2019, at Ras Ijerri. It is a small village in the Saïs plateau in Morocco (33.784098 N–5.710920 W and 598 m of altitude). This area is a well-known zone for onion growing because of its water abundance, favorable edaphic characteristics, and advanced growing techniques. Planting started in the second week of November 2018 with a broadcast sowing of 11 onion ecotype seeds, in a randomized complete block design with three replicates, laid out in 33 experimental units of 1 m^2^. Four months later, only onion plantlets with a good vegetative appearance were transplanted into a new unit of 2 m^2^ in randomized complete blocks with three replicates. The agricultural practices (fertilization, irrigation, and maintenance) were the same for all units and were according to the crop husbandry practices of the Moroccan farmers. The harvest began in the final week of August 2019. Each experimental unit's onion bulbs were stored in plastic mesh bags with a unique label. All of the onion bags were stored in a shed at Ras Ijerri, away from light, until they were used.

### 2.3. Moisture

30 g of fresh onion bulb was dried in a heated oven at 105°C, until it reached a constant weight. Moisture content was calculated as follows: *C* = WF − WD/WF∗100. WF stands for the onion bulb's fresh weight (g), and WD stands for the onion bulb's dried weight (g).

### 2.4. Bioactive Extraction

From each onion ecotype, 500 g of fresh bulbs was cut into thin layers and dried in a ventilated oven (BINDER, Classic line, serial M 115), under 45°C for 24 h, to obtain a final moisture content of 6% (wet basic). To extract the biochemical compounds, 3 g of dried bulb layers was mixed with 30 mL of methanol 80%, under agitation (250 rpm) for 3 h at room temperature, followed by centrifugation (4000 g) at 4°C for 10 min. The supernatant was then filtered through Whatman N°4 paper, and the solution was kept at -20°C in an opaque flask until analysis. Except for the sulfur dioxide content (which was repeated twice), all biochemical compounds were analyzed in triplicate and expressed as dry weight.

### 2.5. Polyphenols

Total phenol content (TPC) was measured by using the Folin-Ciocalteu method. 0.1 mL of the extracted solution was added to 0.5 mL of Folin-Ciocalteu solution ×10. After 15 min, the solution was mixed with 2 mL of sodium carbonate (20%) and left standing for 90 min. As for the absorbance, it was measured by a spectrophotometer (V630 UV-VIS, JASCO) at 790 nm. The gallic acid was used as a standard, and the results were expressed as mg equivalent gallic acid/g dry weight (mg GAE/g DW).

### 2.6. Flavonoids

Total flavonoid content (TFC) was measured according to the Sharma et al. [17] protocol. 0.5 mL of onion extract was mixed with 1.5 mL methanol (80%), 0.1 mL of aluminum chloride (10%), 0.1 mL of potassium acetate (0.1 mol/L), and distilled water (2.8 mL). The solution was kept for 30 min at room temperature. Optical density was measured at 415 nm. Quercetin was used as a standard, and the TFC was expressed in mg equivalent quercetin/g dry weight (mg quercetin E/g DW).

### 2.7. Antioxidant Activity

The antioxidant properties of Moroccan onion ecotypes were determined using the Wang et al. [18] method. Therefore, 3 mL of DPPH (4 mg/100 mL of methanol 80%) was added to 1 mL of onion extract. The solution was retained in a dark place for one hour at room temperature. The absorbance was recorded at 517 nm with methanol as blank and ascorbic acid as standard. The inhibitory concentration (IC50) is the amount of antioxidant required to reduce 50% of the initial DPPH concentration. It was expressed in terms of onion extract concentration (mg/mL). The lower IC50 value indicates high antioxidant activity, whereas the higher IC50 value indicates low antioxidant activity.

### 2.8. Total and Reducing Sugars

Reducing sugar content was determined using the DNS reagent, following the Miller [19] method. The measurement began with DNS preparation, which included adding 1.87 mg of 3,5-dinitro salicylic acid and 3.48 mg NaOH to distilled water. The first solution was then supplemented with 53.9 mg of sodium potassium tartrate, 1.34 mL of phenol, and 1.46 mg of Na metabisulfate. Distilled water was used to make up to 250 mL of the final solution. To quantify reducing sugars, 3 mL of DNS and 2 mL of distilled water were added to each tube containing 1 mL of onion extract. All the tubes were placed in boiling water for 5 min and cooled at room temperature. The optical absorbance was determined at a 575 nm spectrophotometer.

Concerning total sugars, the contents were measured using the DuBois et al. [20] method. In a test tube, 1 mL of phenol (5%) was added to 2 mL of onion extract, followed by 5 mL of sulfuric acid (80%). After allowing the tubes to stand for 10 minutes, they were vortexed for 30 seconds and placed for 15 min in water bath. A spectrophotometer was used to measure absorbance at 490 nm.

### 2.9. Sulfur Dioxide

The Monier-Williams distillation method was used to determine the sulfur dioxide (SO_2_) content of onion ecotype bulbs. However, a minor revision was carried out by collecting the SO_2_ gas in two distillate tubes rather than one; the details of the analysis were given by Türkyılmaz et al. [21, 22]. For each sample, the analysis was repeated twice, and the SO_2_ content was expressed as mg SO_2_/kg dry weight.

### 2.10. Statistical Analysis

The experimental data were statistically analyzed using Minitab18 (Minitab Inc, State College, PA, USA) and NCSS (NCSS CLL, Kaysville, USA). Results were subjected to variance analysis (ANOVA) and Tukey test. The least significant differences were obtained using an LSD test (*p* ≤ 0.05).

The mean content of flavonoids, polyphenol, and DPPH scavenging percentage of each ecotype was calculated to study the correlation level between flavonoids and polyphenols of Moroccan onion with their DPPH scavenging percentage. The analysis correlates each ecotype's flavonoids content with its DPPH scavenging percentage.

## 3. Results

### 3.1. Moisture

Moroccan onion ecotypes showed a minimum and maximum moisture value of 87.80% and 92.30%, respectively (Table 2). ANOVA analysis revealed that there was no statistical difference in the moisture content of the onion bulbs studied. The moisture content of red onions varied between 87.80% and 92.03% depending on the color of the bulb skin. The moisture content of yellow onions ranged from 89.15% to 91.47%. Thus, white onions appear to have the highest moisture content (92.30%), followed by yellow and red onions.

### 3.2. Polyphenols

The total phenol contents (TPC) of eleven onion ecotypes range from 5.94 to 11.22 mg GAE/g DW when measured using the Folin-Ciocalteu method. According to Table 3, red outer skin ecotypes (9.03–11.22 mg GAE/g DW) have a higher concentration of phenolic compounds than yellow (6.10–8.93 mg GAE/g DW) and white (5.94 mg GAE/g DW) ecotypes. According to one-way ANOVA analysis, there was a highly significant difference in phenolic compounds between the eleven onion ecotypes (*p* value < 0.01).

### 3.3. Flavonoids

The flavonoid content of the onion ecotypes studied ranged from 0.67 to 1.52 mg quercetin E/g DW (Table 3). The white onion ecotype contains 0.67 mg of quercetin E/g DW. Red and yellow onions, on the other hand, contain 1.06 to 1.52 mg quercetin E/g DW and 0.81 to 1.02 mg quercetin E/g DW, respectively. The one-way ANOVA analysis revealed the presence of a significant variation between onion ecotypes in terms of flavonoid quantities (*p* value < 0.01). In addition, flavonoid contents among the red and yellow onion ecotype groups are not statistically different (*p* value = 0.341 and 0.08 for red and yellow onions, respectively).

### 3.4. Antioxidant Activity

The antioxidant properties of Moroccan onion ecotypes were evaluated using the DPPH method. Table 3 shows that the free radical scavenging activity of onion ecotypes fluctuates between 0.0989 and 1.153 mg ascorbic acid E/g DW (P2 and P17, respectively). According to these findings, ecotypes P3, P4, P10, P16, and P17 (red bulb color) and P7 and P9 (yellow bulb color) have the highest antioxidant activity when compared to the other onions studied.

Moreover, the antioxidant activity was expressed in terms of inhibitory concentration required to scavenge 50% of free radicals (IC50). Onion IC50 ranged from 0.187 to 1.263 mg/mL of the sample extract (Table 4). Red onion ecotypes presented the highest antioxidant properties with the lowest IC50 value, in comparison to other onion ecotypes. However, the lowest IC50 value indicates a high scavenging ability, whereas the highest value indicates a low antioxidant activity. Furthermore, the one-way ANOVA revealed a highly significant difference between the antioxidant activities of the studied onion ecotypes (*p* value < 0.01).

**Figure 1:**
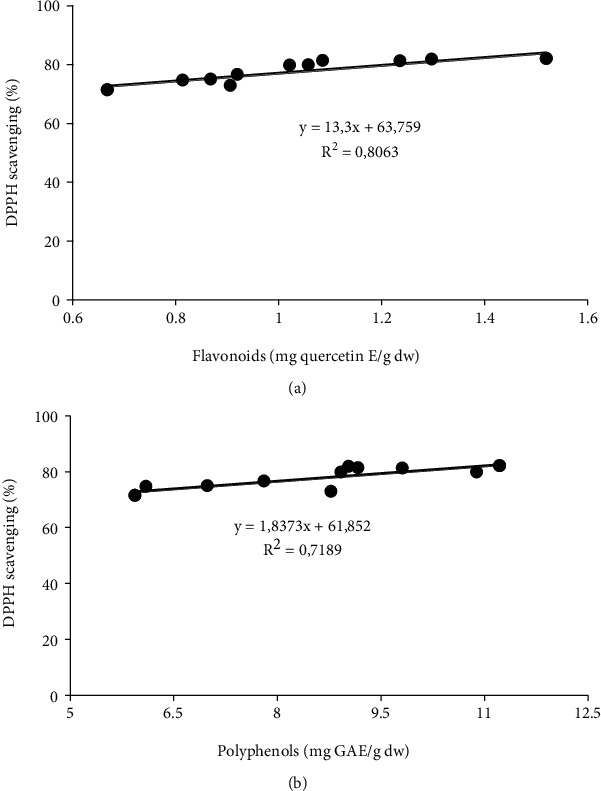
Correlation representation between DPPH scavenging percentage and flavonoids (a) and polyphenols (b).

The correlation between flavonoids and polyphenols with the percentage of antioxidant activity was also determined. With a *p* value < 0.01, the antioxidant percentage correlation with flavonoids (*R*^2^ = 0.8063) (Figure 1(a)) was stronger than the correlation with polyphenols (Figure 1(b)) (*R*^2^ = 0.7189).

### 3.5. Total Sugars

Ecotypes P8 (26.36%) and P3 (64.01%) contain the maximum and the minimum total sugar value, respectively (Table 5). Based on bulb skin coloration, the white onion ecotype contains 31.09% of total sugars. For the red and yellow onion groups, the total sugar values ranged between 31.11 and 64.01% and 26.36 and 42.19%, respectively. ANOVA analysis revealed a highly significant variation between all onion ecotypes studied. In addition, a significant variation was detected between red onion ecotypes (*p* value < 0.01). Tukey's test grouped the red onion ecotypes as follows: P3–P10, P10–P4, P4–P16, and P16–P17. In contrast, the total sugar content among the yellow onion group was not statistically different.

### 3.6. Reducing Sugars

Reducing sugar contents were evaluated using the DNS method (Table 5), and it ranged between 12.05% and 34.73%. Depending on the color of the bulb skin, red onions contain between 22.01 and 34.73% of reducing sugars. The reducing sugar content of yellow onions ranged from 22.24 to 32.02%, while white onions contain 12.05%. Hence, a high significant difference was detected between the onion ecotypes examined in terms of reducing sugar content (*p* value < 0.01). Additionally, inside the red and yellow onion groups, a significant variation was also present, with a *p* value of 0.021 and *p* ≤ 0.001 for the yellow and red groups, respectively.

### 3.7. Sulfur Dioxide

**Figure 2:**
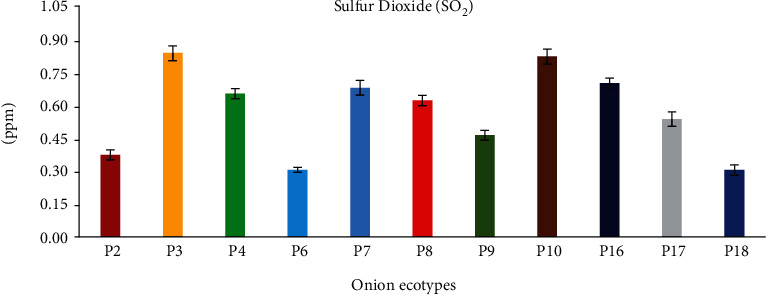
Sulfur dioxide content of eleven Moroccan onion ecotypes (g SO_2_/kg DW).

Figure 2 depicts the sulfur dioxide content of onion bulbs. P3 and P18 contain the highest (85.69 ppm) and lowest (30.429 ppm) sulfur dioxide levels, respectively. The SO_2_ content in red and yellow onion ecotypes ranged from 53.65 to 85.69 ppm and 30.42 to 69.66 ppm, respectively, while the content in white onion ecotype was 38.43 ppm. Also, a highly significant difference was detected between the onion ecotype contents, with a *p* value < 0.01.

## 4. Discussion

The onion plant has been always an important element in the human diet. It offers strong health-promoting effects, including anticarcinogenic properties, antithrombotic activity, antiasthmatic, and antibiotic effects [4, 5]. To ensure a high production level, onion growers resorted to intensification culture practices, specially adapted to hybrids and pure lines of onions. These actions reduce the abundance of traditional landraces and ecotypes, which could be rich resources of interest traits to face future problems especially climate changes [23]. The characterization of the biochemical diversity of Moroccan ecotypes will contribute in the promotion and preservation of these important resources. The present study addresses for the first time in Morocco a biochemical characterization of eleven onion ecotypes, collected from different regions but cultivated under the same environment, to avoid the environment effect on the expression of biochemical characters.

Onion moisture is widely variable around the world. It is affected by plant genotype and also environmental conditions. In Morocco, onion moisture can reach a value between 87.80% and 92.30%. However, according to several studies, onion moisture was estimated between 70% and 90% [24, 25].

The measurement of phenolic content for eleven onion ecotypes, using the Folin-Ciocalteu method, showed a significant difference between the studied onions which ranged from 5.94 to 11.22 mg GAE/g DW. Moreover, several studies conducted on the phenolic content of onions revealed a larger variation in the TPC value among onion landraces. TPC of Italian onion cultivars ranged from 3.9 to 8.2 mg GAE/g [26]. According to Mitrová et al. [27], onion cultivars in the Czech Republic presented a significant difference in polyphenol content, which varied between 2.66 and 3.37 mg GAE/g DW. Likewise, the TPC of 18 onion cultivars in the Republic of Korea altered between 2.95 and 5.544 mg GAE/g DW [28]. Other phenolic concentrations have been reported from different studies, like 25.96 mg GAE/g DW [29]. Further to that, the current study found that, based on bulb skin coloration, white onion had the lowest TPC level compared to red and yellow bulbs, which matched the findings of Lee et al. [8]. In contrast, Zhang et al. [30] found that the yellow onion cultivar in China contained the higher TPC value, followed by the red and white cultivars.

Onions' main phenolic compounds are flavonoids. Flavonols and anthocyanins are the two main subclasses found in *Allium cepa* L. The average flavonoid content in the eleven Moroccan onion ecotypes ranged from 0.67 to1.52 mg quercetin E/g DW. Other flavonoid values in onion cultivars around the world were also reported in recent studies, like 449.75 *μ*g/g FW [13], 415–1917 mg/kg FW of flavonols for red onion, and 270–1187 mg/kg FW for yellow onion [31]. It was also noted that flavonoid concentration in the onion bulb decreased from the outer to the inner onion layers [11]. Previous research revealed that onion with red skin color contains more flavonoids than yellow or white color onion [11, 30]. According to the findings of the present study, the concentration of flavonoids in white onion ecotypes was lower than in red or yellow onion bulbs. Similarly, Pérez-Gregorio et al. [12] reported that the flavonol content in white onion was also lower compared to red onion cultivars. In contrast, Zhang et al. [30] reported an absence of flavonoids in white onion cultivars in China. However, the statistical analysis confirmed that the flavonoid contents in the red onion ecotype were completely different from that of the yellow ecotypes. On the other hand, the flavonoid contents among red onion groups were statistically similar. The same results were detected among yellow onions.

The loss of violet coloration could be used to determine the scavenging of free radical DPPH by a substrate, due to the donation of a hydrogen atom. The antioxidant potential of Moroccan onion ecotypes was estimated at 0.0989 and 1.15 mg ascorbic acid E/g DW, which is higher than the antioxidant activity of the Czech Republic onion [27]. For the Moroccan red onion ecotype, it is enough to add 0.187 to 0.404 mg/mL to reduce 50% of the DPPH free radicals, but for the white ecotype, it is necessary to add 1263 mg/mL. In general, the antioxidant properties of Moroccan onion ecotypes increase from white onions to yellow and red onions. Furthermore, two Moroccan yellow onion ecotypes exhibited antioxidant properties similar to the five red onions studied. However, according to the findings of Zhang et al. [30], the antioxidant activity of Chinese onions varied depending on the color of the bulb skin. Since then, red onion cultivars in China have demonstrated the highest antioxidant activity when compared to yellow and white cultivars. As a result, the antioxidant properties of onions are determined by factors other than the color of the bulb skin. The scavenging of 50% of DPPH free radicals by onion extract varied between 0.19 and 0.24 mg/mL for the bulb peel in an in vitro test [32]. Several standards could be used to report on the antioxidant activity of DPPH in onion such as gallic acid (500–750 mg GAE/mL) [8], ascorbic acid (5–40.1 *μ*mol/g) [33], or trolox (15.08–22.90 *μ*mol trolox E/g DW) [14].

A good linear correlation was detected between the flavonoids and polyphenols with antioxidant activity, *R*^2^ = 0.8063 and 0.7189, respectively. This proves that the antioxidant activity was strongly linked to polyphenol content, especially the flavonoids [9, 34, 35]. Further correlation studies showed that the antioxidant activity correlated strongly with the flavonoids (*R*^2^ = 0.826), in comparison with polyphenols (*R*^2^ = 0.629) [11]. Sharma et al. [28] reported a positive correlation between the antioxidant activity with polyphenols (*R*^2^ = 0.7755) and with flavonoids (*R*^2^ = 0.7137). However, Zhang et al. [30] reasoned that even with the absence of flavonols, white onion cultivars still possess a good antioxidant activity. In addition, Sellappan and Akoh [36] noted a very weak correlation between polyphenols with antioxidant activity (*R*^2^ = 0.04), and *R*^2^ = 0.34 with flavonoids.

The total and reducing sugar contents of the Moroccan onions studied showed a high significant and different concentration within the red onion group, with the five ecotypes significantly different in terms of total and reducing sugar contents. On the other hand, yellow onions differ only in terms of reducing sugar content, while the white onion ecotype had the lowest reducing sugar content. Thus, white onions are recommended by processing units to avoid caramelization during dehydration due to their low reducing sugar content [37].

Sugar contents are affected essentially by temperature. That is, during plant development, air temperature affects the accumulation of sugars in the onion bulb, where a temperature of up to 25°C stimulates the concentration of sugars, which generates a strong sweet flavor [38]. Besides, during onion processing, the sugar content decreases with the increase of the temperature (over 100°C) [17]. Carbohydrates constitute a large part of onion bulb dry weight [17], which explains the high sugar content in dry weight compared to fresh weight [25]. Moreover, onion bulbs in Burkina Faso presented higher total and reducing sugar contents [39] compared to our study. However, red onion groups presented a high total sugar level compared to the rest of the onions studied, which is confirmed by Kandoliya et al. [6]. Ecotype P3 contains more total sugars than P9, but the reducing sugar content of P9 is higher than that of P3. That affirms the absence of correlation between the total and reducing sugar contents [39].

With regard to the measurement of sulfur dioxide, Monier-Williams' method is considered the official analytical procedure for the Food Code in Korea [40]. The sulfur dioxide detected by Monier-Williams' method in onions could come from natural sulfur dioxide content or from some natural sulfur components, which are able to have a similar reaction like natural sulfur dioxide [41]. There is a scarcity of research focusing on the quantitative measurement of sulfur dioxide in onions. In 1927, Monier-Williams had reported for the first time sulfur dioxide content in fresh onion (1 ppm). For dried onion, Dyer et al. [41] have found that sulfur dioxide content ranged between 25 and 70 ppm. The current results revealed the same average. Moroccan onions have a sulfur dioxide content that ranges between 30.43 and 85.60 ppm. Other sulfur dioxide contents were reported in onion, including 75.4 ppm in dried onion [42] and 10.60 ppm in fresh onion found by Kim and Lee [40].

According to Dyer et al. [41], the high content of sulfur dioxide in dried onions could have been caused by a natural content of SO_2_ in onion or by natural sulfur containing the ability to react like sulfur dioxide in the Monier-Williams method.

Sulfur dioxide is a generally harmless preservative that is used to extend the shelf life of products by inhibiting the germination of microorganisms [43]. In addition, by protecting compounds from oxidation, sulfur dioxide preserves their organoleptic properties [44]. However, the high content of natural sulfur dioxide in onions ensures good and long-term storage of fresh and dried onions without the need for synthetic sulfur dioxide.

## 5. Conclusion

The study of the biochemical properties of eleven Moroccan onion ecotypes confirms that the onions studied contain a high level of phenolic components, particularly the flavonoids, which explains the high antioxidant activity detected. Despite the fact that some of the studied onion ecotypes originated from the same geographic region, significant diversity was found in the studied characters, with the exception of the moisture contents, which were statistically identical. This study presents, for the first time, the biochemical profiles of red, yellow, and white onion ecotypes in Morocco. It also allows for the characterization of the current diversity among onions under the same environment. These findings are essential for the introduction of newly adapted ecotypes into the Ras Ijerri environment, as well as for any future valorization programs.

## Figures and Tables

**Table 1:** Geographic origin, altitude, annual temperature, and annual rainfall of collected onion ecotype seeds.

Ecotype	Origin	Skin color	Altitude (m)	Annual temperature (°C)	Annual rainfall (mm)
P2	Zaouit Echikh	White	1442	13–32	40.5
P3	Souk Sabt	Red	421	11–32	38.5
P4	Tetouane	Red	192	13–27	30.8
P6	Doukkala	Yellow	61	15–26	32.0
P7	El Gara	Yellow	305	13–31	30.4
P8	Berrechid	Yellow	230	14–30	29.7
P9	Agourai	Yellow	894	12–32	58.8
P10	Agourai	Red	894	12–32	58.8
P16	Goulmima	Red	1018	10–35	20.7
P17	Ait Malek	Red	1195	13–33	21.9
P18	Bouderbala	Yellow	768	7–24.5	57.2

**Table 2:** Moisture content of eleven Moroccan onion ecotypes (%).

Ecotype	P2	P3	P4	P6	P7	P8	P9	P10	P16	P17	P18
Moisture (%)	92.30 ± 1.81	90.5 ± 1.30	92.0 ± 2.23	90.8 ± 2.23	91.4 ± 2.93	89.2 ± 0.96	91.2 ± 1.79	91.1 ± 0.49	90.8 ± 1.62	87.8 ± 0.69	91.5 ± 0.58

**Table 3:** Polyphenols, flavonoid contents, and antioxidant activity of eleven Moroccan onion ecotypes.

Ecotype	P2	P3	P4	P6	P7	P8	P9	P10	P16	P17	P18
Polyphenols (mg GAE/g DW)	5.94 ± 0.65^e^	9.81 ± 0.61^ab^	9.17 ± 0.22^ab^	6.99 ± 0.69^de^	8.78 ± 0.27^cd^	6.10 ± 0.8^e^	8.93 ± 0.86^bc^	10.89 ± 0.81^ab^	9.03 ± 0.81^bc^	11.22 ± 0.96^a^	7.81 ± 0.62^cd^
Flavonoids (mg quercetin E/g DW)	0.67 ± 0.07^c^	1.24 ± 0.02^ab^	1.09 ± 0.07^ab^	0.87 ± 0.05^b^	0.91 ± 0.07^b^	0.81 ± 0.07^b^	1.02 ± 0.01^ab^	1.06 ± 0.02^ab^	1.30 ± 0.04^ab^	1.52 ± 0.04^a^	0.92 ± 0.08^b^
Antioxidant activity (mg ascorbic acid E/g DW)	0.9 ± 0.05^b^	1.13 ± 0.04^a^	1.13 ± 0.03^a^	1.05 ± 0.04^ab^	1.12 ± 0.06^a^	1.05 ± 0.01^ab^	1.12 ± 0.01^a^	1.12 ± 0.01^a^	1.15 ± 0.02^a^	1.15 ± 0.01^a^	1.08 ± 0.01^ab^

Data are expressed as means ± SE of triplicate analyses of each triplicate extraction. Values in the same row with different letters present significant differences, *p* value < 0.01.

**Table 4:** DPPH free radical scavenging activity, expressed in IC50 (mg/mL).

Ecotype	P2	P3	P4	P6	P7	P8	P9	P10	P16	P17	P18
IC50 (mg/mL)	1.263 ± 0.063^i^	0.378 ± 0.019^c^	0.295 ± 0.014^b^	0.684 ± 0.034^g^	0.438 ± 0.021^e^	1.055 ± 0.052^h^	0.420 ± 0.021^e^	0.404 ± 0.02^d^	0.216 ± 0.011^a^	0.187 ± 0.009^a^	0.568 ± 0.028^f^

Data are expressed as means ± SE of triplicate analyses of each triplicate extraction. Values in the same row with different letters present significant differences, *p* value < 0.01.

**Table 5:** Total and reducing sugar content of eleven Moroccan onion ecotypes (g glucose/100 g DW).

Ecotype	P2	P3	P4	P6	P7	P8	P9	P10	P16	P17	P18
Total sugars (%)	31.09 ± 1.39^e^	64.0 ± 1.28^a^	42.86 ± 0.53^bc^	38.06 ± 1.57	29.87 ± 1.68	26.36 ± 1.59	39.22 ± 1.06	50.2 ± 1.20^ab^	33.9 ± 1.23^cd^	31.11 ± 0.82^d^	42.19 ± 0.95
Reducing sugars (%)	12.05 ± 0.17^f^	24.94 ± 0.92^de^	28.11 ± 0.83^b^	22.24 ± 1.44^e^	24.94 ± 0.95^de^	25.10 ± 0.66^cd^	32.02 ± 0.30^a^	34.7 ± 0.49^a^	22.01 ± 1.08^de^	22.56 ± 0.38^de^	27.41 ± 1.06^bc^

Data are expressed as means ± SE of triplicate analyses of each triplicate extraction. Values in the same row with different letters present significant differences, *p* < 0.01.

## Data Availability

The data used to support the findings of this study are included within the article.
